# *Onchocerca volvulus* transmission in the Mbam valley of Cameroon following 16 years of annual community-directed treatment with ivermectin, and the description of a new cytotype of *Simulium squamosum*

**DOI:** 10.1186/s13071-021-05072-y

**Published:** 2021-11-02

**Authors:** Adam Hendy, Meryam Krit, Kenneth Pfarr, Christine Laemmer, Jacobus De Witte, Philippe Nwane, Joseph Kamgno, Hugues C. Nana-Djeunga, Michel Boussinesq, Jean-Claude Dujardin, Rory Post, Robert Colebunders, Sarah O’Neill, Peter Enyong, Alfred K. Njamnshi

**Affiliations:** 1 Department of Biomedical Sciences, Institute of Tropical Medicine, Antwerp, Belgium; 2 Department of Pathology, University of Texas Medical Branch, Galveston, TX USA; 3 Institute for Medical Microbiology, Immunology and Parasitology, University Hospital Bonn, Bonn, Germany; 4 German Center for Infection Research, Partner Site Bonn-Cologne, Cologne, Germany; 5 Centre for Research on Filariasis and Other Tropical Diseases (CRFilMT), Yaoundé, Cameroon; 6 Department of Public Health, Faculty of Medicine and Biomedical Sciences, The University of Yaoundé I, Yaoundé, Cameroon; 7 Neuroscience Lab, Faculty of Medicine and Biomedical Sciences, The University of Yaoundé I, Yaoundé, Cameroon; 8 Institut de Recherche pour le Développement (IRD), Montpellier, France; 9 Department of Disease Control, London School of Hygiene and Tropical Medicine, London, UK; 10 School of Natural Sciences and Psychology, Liverpool John Moores University, Liverpool, UK; 11 Global Health Institute, University of Antwerp, Antwerp, Belgium; 12 Department of Public Health, Institute of Tropical Medicine, Antwerp, Belgium; 13 CR 5, Ecole de Santé Publique, Université Libre de Bruxelles, Brussels, Belgium; 14 Research Foundation in Tropical Diseases and Environment, Buea, Cameroon; 15 Brain Research Africa Initiative (BRAIN), Yaoundé, Cameroon; 16 Brain Research Africa Initiative (BRAIN), Geneva, Switzerland; 17 Neurology Department, Central Hospital Yaoundé, Yaoundé, Cameroon

**Keywords:** Onchocerciasis, Elimination, Ivermectin, *Onchocerca volvulus*, *Simulium damnosum*, *Simulium squamosum*, Mbam, Cameroon

## Abstract

**Background:**

The onchocerciasis focus surrounding the lower Mbam and Sanaga rivers, where *Onchocerca volvulus* is transmitted by *Simulium damnosum* s.l. (Diptera: Simuliidae), was historically the largest in the southern regions of Cameroon. Annual community-directed treatment with ivermectin (CDTI) has been taking place since 2000, but recent studies have shown that new infections are occurring in children. We aimed to investigate blackfly biting and *O. volvulus* transmission rates along the lower Mbam river 16 years after the formal onset of annual CDTI.

**Methods:**

Black flies were collected for three consecutive days each month between July 2016 and June 2017 at two riverside villages and two inland sites situated 4.9 km and 7.9 km from the riverside. Specimens collected at each site were dissected on one of the three collection days each month to estimate parity rates and *O. volvulus* infection rates, while the remaining samples were preserved for pool screening.

**Results:**

In total, 93,573 *S. damnosum* s.l. black flies were recorded biting humans and 9281 were dissected. Annual biting rates of up to 606,370 were estimated at the riverside, decreasing to 20,540 at 7.9 km, while, based on dissections, annual transmission potentials of up to 4488 were estimated at the riverside, decreasing to 102 and 0 at 4.9 km and 7.9 km, respectively. However, pool screening showed evidence of infection in black flies at the furthest distance from the river. Results of both methods demonstrated the percentage of infective flies to be relatively low (0.10–0.36%), but above the WHO threshold for interruption of transmission. In addition, a small number of larvae collected during the dry season revealed the presence of *Simulium squamosum* E. This is the first time *S. squamosum* E has been found east of Lake Volta in Ghana, but our material was chromosomally distinctive, and we call it *S. squamosum* E2.

**Conclusions:**

Relatively low *O. volvulus* infection rates appear to be offset by extremely high densities of biting black flies which are sustaining transmission along the banks of the lower Mbam river.

**Graphical Abstract:**

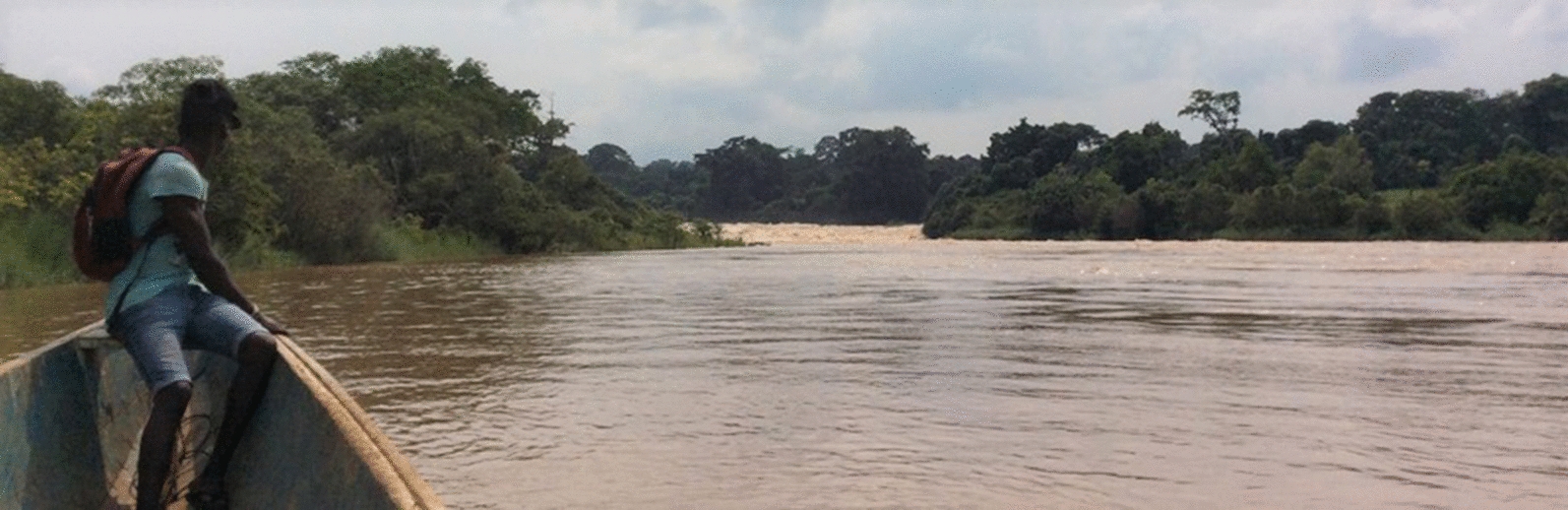

**Supplementary Information:**

The online version contains supplementary material available at 10.1186/s13071-021-05072-y.

## Background

Onchocerciasis was hyperendemic in five main foci in Cameroon before community-directed treatment with ivermectin (CDTI) was introduced to control the disease in 1997 [1, 2]. The foci extended across savannahs in the northern regions, through forest–savannah transition zones in the centre, and into dense humid forests further south [1, 3]. Fast-flowing rivers running through these areas provide suitable breeding habitats for *Simulium damnosum* (Diptera: Simuliidae) complex black flies, the only vectors of *Onchocerca volvulus* in the country [4, 5].

Eight cytoforms (both cytospecies and cytotypes) of the *S. damnosum* complex have been described from Cameroon [5], and their geographical distribution has been well mapped [4, 6]. *Simulium damnosum *sensu stricto (s.s.) and *Simulium sirbanum* are cytospecies common in savannah habitats [6], while *Simulium yahense* and *Simulium squamosum* are associated with forest and transitional zones [4, 6], although the latter may spread north into savannah rivers during the rainy season [4]. *Simulium squamosum* is divided into five cytotypes (chromosomally distinct populations of unconfirmed taxonomic status) designated A–E, of which cytotypes A–D are present in Cameroon, while cytotype E occurs west of Lake Volta in Ghana [4, 7, 8]. *Simulium squamosum* A is the typical form described by Vajime and Dunbar [9]. It is found throughout most of Cameroon except for the Sanaga river, which is the only known breeding locality of *S. squamosum* B [4]. Cytotypes C and D are known from the areas around Mount Cameroon where A and C also appear to interbreed [4, 7]. Another member of the complex, *Simulium mengense*, has a scattered distribution throughout the north where it occupies similar habitats to *S. damnosum* s.s. and *S. sirbanum* [6]. It is also present in rivers around Mount Cameroon and in the Centre Region, where it often breeds sympatrically with *S. squamosum* [10–12]. All *S. damnosum *sensu lato (s.l.) cytospecies present in Cameroon are known or suspected vectors of *O. volvulus* [13–15].

The onchocerciasis focus surrounding the lower Mbam and Sanaga rivers was historically the largest in the southern regions of Cameroon [14]. Disease prevalence was particularly high in villages along the lower Mbam, where infection was associated with severe ocular pathologies and high rates of epilepsy [2, 11, 16]. The latter condition was found to be related to high community microfilarial loads following a study by Boussinesq et al. in 1991/1992 [16]. Furthermore, two cohort studies in these river valleys have shown a temporal relationship with a dose effect between microfilarial load in children and the development of epilepsy [17, 18]. Prior to the implementation of CDTI, an investigation of blackfly biting rates and *O. volvulus* transmission was conducted by Barbazan et al. [11]. This took place over 12 months from April 1993 until March 1994 and included two transects perpendicular to the Mbam river and close to the town of Bafia. The study showed that blackfly biting rates were high throughout the year, but *O. volvulus* transmission was seasonal, occurring mainly between February and May and peaking in February and March. Breeding populations of *S. damnosum* s.l. were restricted to the main river, where 90% of larvae were found to be *S. squamosum* s.s. (presumably cytotype A) and 10% were *S. mengense* [11].

There is no history of vector control along the Mbam river, where onchocerciasis is controlled solely through annual CDTI [3, 19]. The first large-scale ivermectin treatments commenced in 1994 in selected villages as part of a clinical trial to evaluate the macrofilaricidal potential of the drug [11, 20]. CDTI initially commenced in 1997 but encountered problems due to severe adverse events associated with *Loa loa* co-infection [2]. Treatment coverage during the first years was consequently low, and the programme was relaunched in 2000 [2, 3]. Epidemiological studies conducted in 2011 showed that the CDTI project, Centre 1, which includes Bafia Health District, had a mean microfilarial prevalence of 52.3% based on a survey of 12 villages, and was progressing more slowly than expected towards elimination [3, 21]. After corrective measures had been made to the CDTI project, Kamga et al. [3] conducted a follow-up survey in 2015 which revealed a mean microfilarial prevalence of 41.6% in four villages. While this was still higher than expected, community microfilarial loads had decreased dramatically from pre-control levels, but microfilaridermia and the presence of nodules in children aged less than 10 years implied that transmission was ongoing [3].

Onchocerciasis is still a country-wide public health problem in Cameroon, and at the onset of this study there was evidence of ongoing *O. volvulus* transmission despite long-term public health interventions [3, 22]. Between July 2016 and June 2017, we investigated blackfly biting rates and seasonal patterns of *O. volvulus* transmission near villages surrounding the lower Mbam river, almost 23 years after the pre-control survey by Barbazan et al. [11] and 16 years after the formal onset of annual CDTI [3, 11].

## Methods

### Study area

**Fig. 1:**
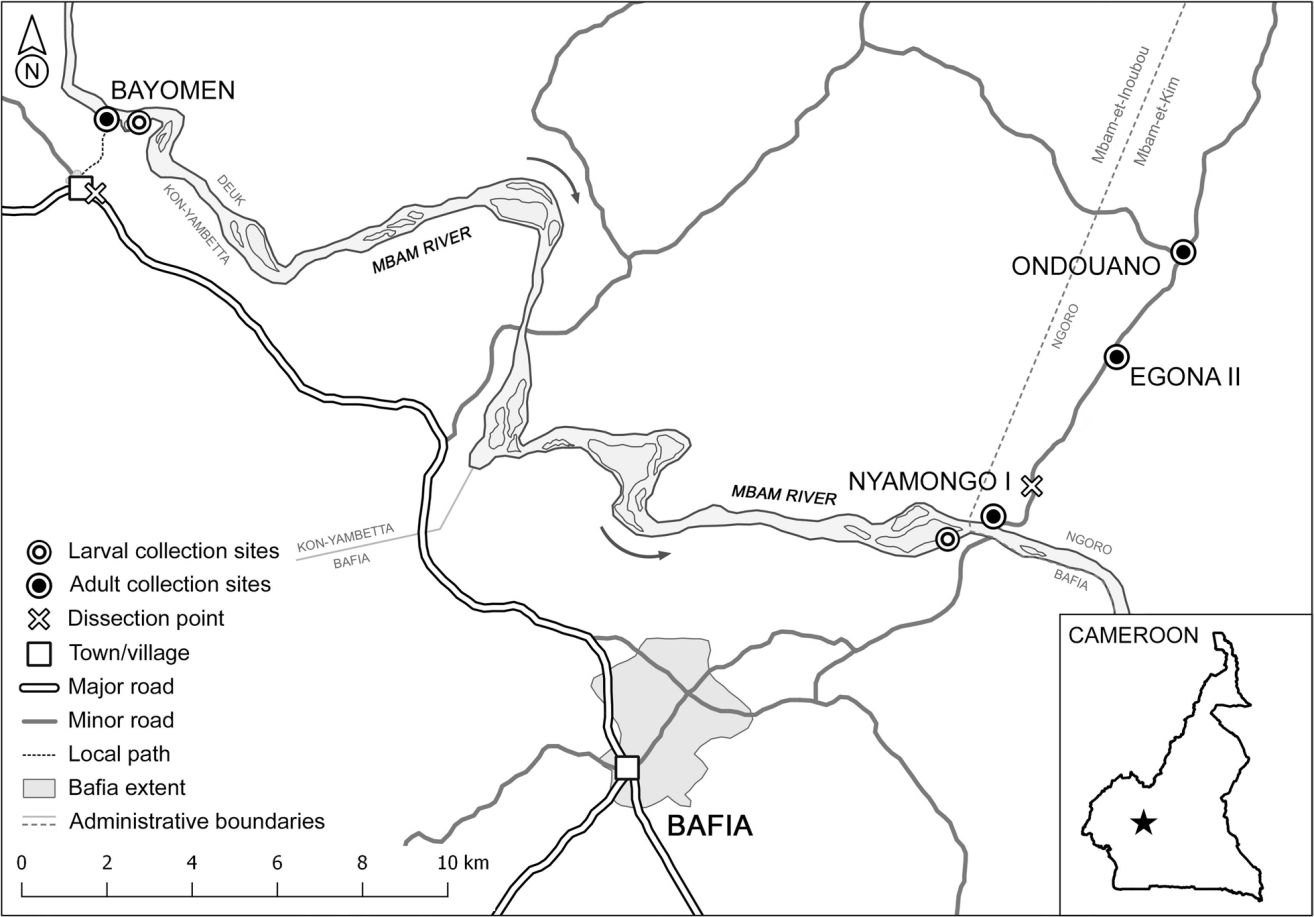
Map of the study area showing adult and larval blackfly collection sites and dissection stations in relation to the Mbam river. Grey arrows show the direction of river flow and the inset map shows the location of the study area (black star) in Cameroon

**Fig. 2:**
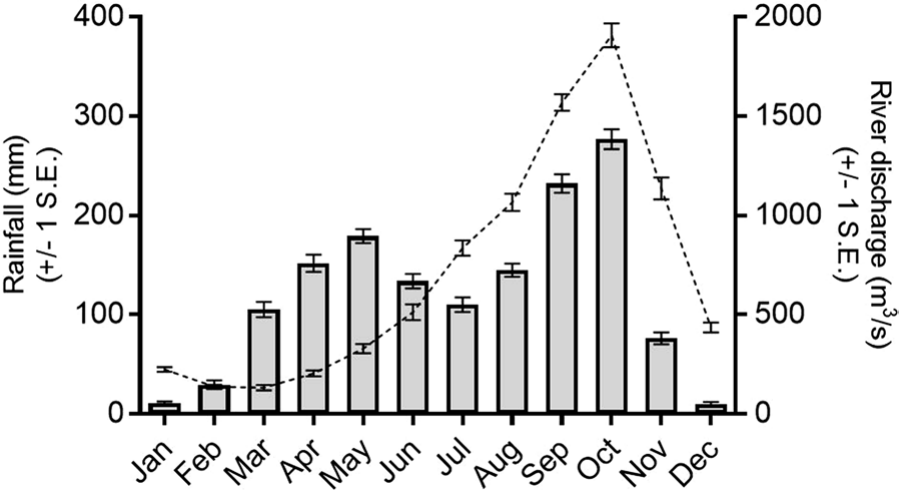
Historical rainfall and river discharge for Bafia and the lower Mbam river. Bars show mean monthly rainfall (mm) ± 1 standard error (SE) at Bafia for years 1930–1994 [26]; dashed line shows mean monthly river discharge (m^3^/s) ± 1 standard error (SE) for years 1952–80 for the Goura gauge located on the Mbam river ≈25 km SE of Bafia (4.56703°N, 11.36740°E) [27]

The study was conducted in villages close to the perennially flowing Mbam river near Bafia (4.73939°N, 11.22073°E) in the Centre Region of Cameroon (Fig. 1). The Mbam originates in the northern savannah region and flows through the transitional forest–savannah mosaic surrounding Bafia before joining the Sanaga river as its main tributary [23]. As it passes near Bafia, the river is characterised by a series of rapids that provide ideal sites for blackfly breeding [16]. The climate is equatorial and the area receives 1700–1850 mm in annual rainfall, occurring mainly in two peaks (Fig. 2) [24]. The lesser peak occurs between March and June which precedes a brief dry period in July and is followed by heavier rainfall between August and October. River discharge steadily increases across the rainy seasons before it abruptly decreases in November at the start of the long dry season (Fig. 2) [11]. blackfly biting occurs throughout the year but is most intense between February and May, and September and October. Transmission of *O.* *volvulus* mainly occurs between February and May [11]. The estimated population of Bafia Health District was 226,073 in 2014 [3]. Many residents engage in subsistence farming, fishing, and sand mining along the Mbam river [3], while nomadic pastoralists (Bororo herdsmen) migrate to the area at varying times between November and May each year (M Ronse pers. comm.). Small numbers of cattle are also kept locally [25].

### Study sites

blackfly collection sites were established at or near four villages along the lower Mbam river (Table 1). The sites along the transect extending from Nyamongo I to Ondouano were chosen to enable comparison with data collected by Barbazan et al. [11], while the site near Bayomen was chosen due to historically high rates of epilepsy in the area [16]. Nyamongo I and Bayomen sites were both at the riverside. The former was next to a ferry crossing approximately 1 km downstream from major rapids, while the latter was used by local fishermen and was known for its high densities of biting black flies. Collection sites at Egona II and Ondouano were located 4.9 km and 7.9 km from the riverside, respectively.

**Table 1:** Villages and collection sites

Village	Population	Collection site coordinates	Distance to river
Bayomen	962	4.87850°N, 11.11140°E	0 km
Nyamongo I	1000	4.79143°N, 11.29647°E	0 km
Egona II	1450	4.82829°N, 11.32183°E	4.9 km
Ondouano	590	4.84976°N, 11.33883°E	7.9 km

Population data obtained from Programme National de Développement Participatif reports [25, 28]

### Collection and preservation of black flies

blackfly larvae and pupae were collected from rocks and trailing vegetation in large rapids 1 km upstream from the Nyamongo I ferry crossing and 300 m downstream from the collection site at Bayomen in January 2017 (dry season), when the river was most accessible (Fig. 1). Samples were placed in three changes of Carnoy’s fixative (≈3:1 ethanol: glacial acetic acid) to preserve *S. damnosum* complex larval polytene chromosomes for cytotaxonomic study. All pupae and non-damnosum larvae were transferred to absolute ethanol in the laboratory, where they were stored at −20 °C until needed.

Adult black flies were collected simultaneously at the four sites for three consecutive days each month, beginning in July 2016 and ending in June 2017. At each site, catches were made between 07:00 and 18:00 daily by two individuals working alternate hours who were recruited from the nearest village and trained in standard human landing collection methods [29]. Due to anticipated high landing rates, black flies were collected using mouth aspirators which were labelled and changed hourly. Adult black flies were either dissected in the field to determine parity rates and to detect *Onchocerca* spp. larvae or were preserved in absolute ethanol for subsequent pool screening. Preserved specimens were maintained at ambient temperatures in the field until stored at 4 °C in the laboratory. Landing rates were interpreted as being representative of exposure to biting and are, therefore, referred to as biting rates.

### Identification of *S. damnosum* complex

Larvae of the *S. damnosum* complex were morphologically identified by the presence of dorsal abdominal tubercles and scales on the prothoracic proleg [30]. Late-instar larvae were used for cytotaxonomy. Prior to chromosome preparation, the head and thorax of each larva was separated from the remainder of the specimen and stored individually in absolute ethanol for morphological identification. Salivary glands were then dissected from the abdominal cavity of each respective specimen and were prepared following a Feulgen staining method outlined by Adler et al. [31]. Larvae were identified with reference to the cytotaxonomic key in Post et al. [15], and chromosome maps in Vajime and Dunbar [9], Boakye [32], and Mustapha et al. [10]. Inversion nomenclature follows Post et al. [33]. Adult black flies were identified by their enlarged fore-tarsi bearing crests of dark hair and the presence of white bands on the hind basi-tarsi [34].

### Dissection of adult black flies

Field stations were established at Bayomen and Nyamongo I (Fig. 1) to estimate *S. damnosum* s.l. parity rates and *Onchocerca* spp. infection rates at each site. To achieve this, a subsample of adult black flies (up to ≈30 per hour, and ≈330 per day) were dissected from each site on one of the three collection days each month. Black flies were exposed to chloroform vapour inside a closed box until anaesthetised. Each specimen was then placed in a drop of saline solution on a microscope slide and dissected to determine whether it was parous or nulliparous using standard methods [35]. Nulliparous flies were discarded while parous flies were further dissected and examined for the presence of *O. volvulus* larvae [35, 36]. If parasites were found, the number and developmental stages (L1–L3, and L3H) were recorded. L3 larvae were defined as infective stages found anywhere within the blackfly, while L3H were those present in the head. Dissections were carried out by experienced technicians using Wild M5 stereo microscopes (Wild Heerbrugg, Switzerland) illuminated with 6v bulbs.

### Pool screening

The objective of pool screening was to confirm the identity of the *Onchocerca* species present in *S. damnosum* s.l. and to provide a robust estimate of infection rates using a larger sample of black flies. Pools of heads and bodies (maximum pool size *n* = 200) of ethanol-preserved black flies were prepared separately, by site and month of collection, following methods outlined by Hendy et al. [37]. DNA was extracted and samples were checked for PCR inhibitors as described previously [38]. Samples positive for PCR inhibitors were serially diluted (1:10 steps) until no inhibition remained. Dilutions of up to 1:100 and 1:10,000 were necessary to remove inhibition in pools of bodies and heads, respectively. Diluted samples were then used in a triplex real-time PCR that differentiates *O. volvulus* from *Onchocerca ochengi*, a morphologically similar bovine parasite also transmitted by *S. damnosum* s.l., based on differences in their respective ND5 genes (GenBank: AY462885.1 and FM206483.1) [37]. Samples were not screened for *Onchocerca ramachandrini*, a parasite of warthogs (*Phacochoerus africanus*) also transmitted by *S. damnosum* s.l. [39], since the study area was beyond the limit of its host range [40].

Triplicate reactions were carried out in 20 μl total volumes containing 2 μl template DNA or water as a negative control, 1× HotStar Taq Buffer (Qiagen, N.V.), 4.5 mM MgCl2, 200 µM dNTPs (50 µM each, Fisher Scientific GmbH, Schwerte, Germany), 0.5 units HotStar Taq (Qiagen), and primers (Microsynth AG, Balgach, Switzerland) and hybridization probes (biomers.net GmbH, Ulm, Germany) at the concentrations listed in Table 2 [37]. The reactions were performed in a Rotor Gene Q cycler (Qiagen, Hilden, Germany) with the following cycling conditions: *Taq* polymerase activation at 95 °C for 15 min and then 45 cycles at 95 °C for 10 s and 61 °C for 30 s, with fluorescence acquisition on the Fam, Hex, and Cy5 channels at the end of each cycle. Plasmids containing the respective sequences were used as PCR-positive controls in every run [38]. Due to the need for such strong dilutions (1:10,000), the samples were run in triplicate up to four times. Samples were accepted as true positives for *O. volvulus* or *O. ochengi* infection if at least two of the three runs were positive, or if one run was *O. volvulus* ND5-positive alongside a positive 16S result (pan-*Onchocerca* probe). Bodies positive for either filarial species were considered to contain microfilariae or developing larvae, while heads positive for either species were considered to contain L3 larvae.

**Table 2:** Triplex PCR primers and hybridization probes for *O. volvulus* (Ov), *O. ochengi* (Oo), and *Onchocerca* spp. (16S), with final concentrations in the real-time PCR runs

Primers	Sequence and modification	Concentration (nM)
OvOo ND5 forward	GCTATTGGTAGGGGTTTGCAT	300
OvOo ND5 reverse	CCACGATAATCCTGTTGACCA	300
Ov probe	Fam-TAAGAGGTTATTGTTTATGCAGATGG-BHQ1	50
Oo probe	Hex-TAAGAGATTGTTGTTTATGCAGATAGG-BHQ1	50
16S rDNA forward	AATTACTCCGGAGTTAACAGG	500
16S rDNA reverse	TCTGTCTCACGACGAACTAAAC	500
16S rDNA probe	Cy5-TACAACATCGATGTAGCGCAGC-BBQ-650	75

### Statistical analyses

The Wilson method was used to calculate confidence intervals for all proportions (biting rates, parity rates, and L1–L2 and L3H infection rates), as recommended by Agresti and Coull [41]. The coverage probabilities were close to the nominal confidence levels. All the confidence intervals used later were calculated at the 95% significance level. Biting rates were analysed using a generalized negative binomial regression to take into account overdispersion and to test possible differences across collection sites and seasons. A logistic regression model was used to test the effect of seasonality on parity and infection rates between the different collection sites. The above analyses were performed in R v4.1.0 [42]. blackfly collection and dissection data were used to estimate monthly and annual *S. damnosum* s.l. biting rates and the monthly (MTP) and annual (ATP) transmission potentials at each site following methods described by Walsh et al. [29]. Poolscreen v2.0 [43] was used to estimate *O. volvulus* infection rates (maximum likelihood) with 95% confidence intervals (Bayes credibility estimate) at each site. Infection rates were only estimated in pools of heads since almost all pools of bodies were positive and the sensitivity of analysis is not known when pool positivity exceeds 25% [43].

## Results

### Identification of *S. damnosum* complex

**Fig. 3:**
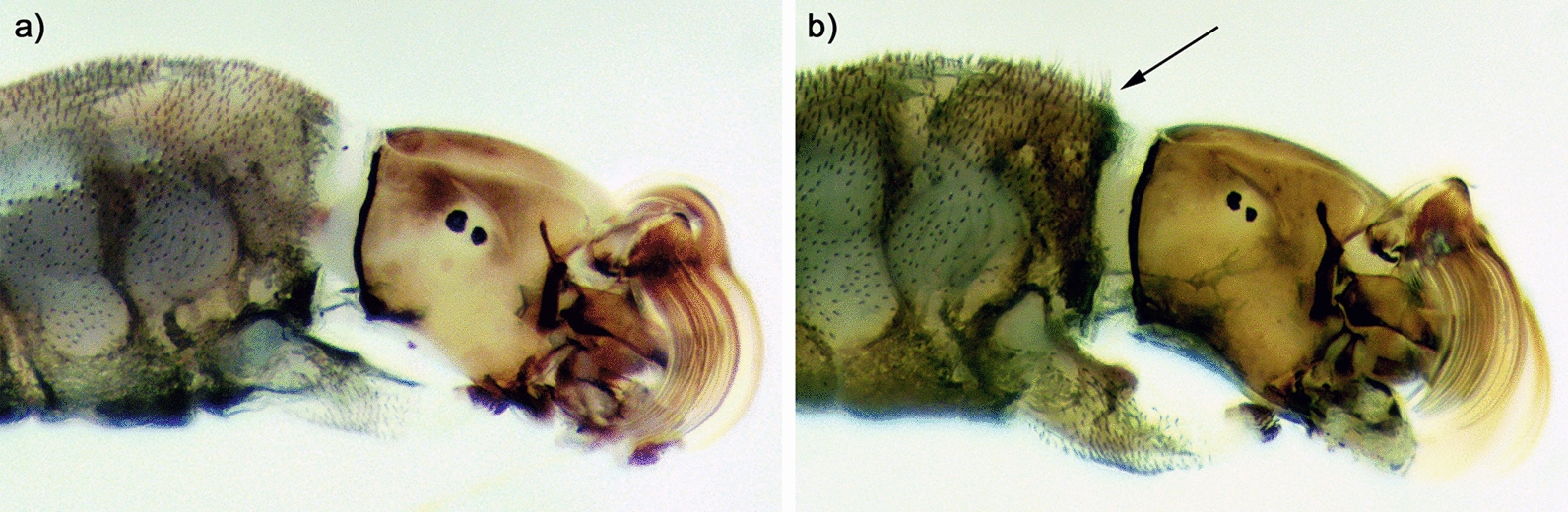
Head and thorax of late-instar larvae showing **a** *S. squamosum* E2, and **b** *S. mengense* with arrow pointing to tuft of hair-like scales on the anterior dorsum of the thorax

**Fig. 4:**
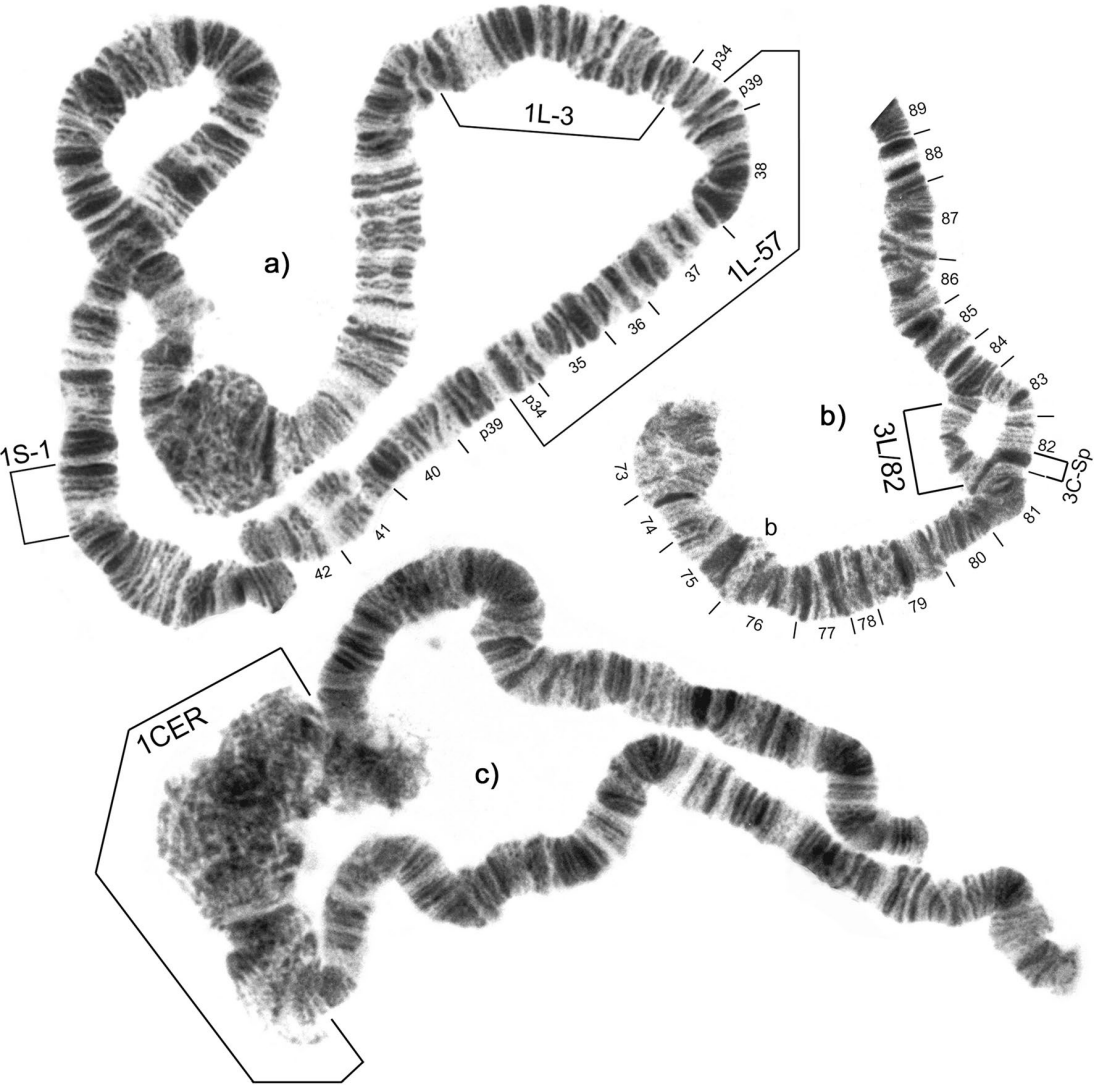
Polytene chromosomes of *S. squamosum* E2 and *S. mengense* showing **a** chromosome 1 of *S. squamosum* E2 with fixed inversions 1S-1 and 1L-3, and inversion 1L-57 which was fixed in the specimens examined, **b** part of chromosome 3 of *S. squamosum* E2 male showing sex-linked band dimorphism 3C-Sp and sex-linked heterozygous inversion 3L/82 present in 19/20 male specimens examined, ‘b’ = blister, and **c** chromosome 1 of *S. mengense* showing expanded centromere 1CER

Morphological and cytotaxonomic identification of *S. damnosum* complex larvae revealed the presence of *S. squamosum* s.l. and *S. mengense* (Figs. 3, 4), both of which have previously been reported from the area [11]. However, *S. squamosum* did not conform to descriptions of the cytotypes A, B, C, or D, known from Cameroon [4, 5, 7]. All specimens analysed from Nyamongo I (*n* = 39) and Bayomen (*n* = 10) contained homozygous inversions 1S-1 and 1L-3, which are generally fixed in the *S. squamosum* subcomplex (Fig. 4a) [32]. They also possessed a new 1L inversion from sections p34 to p39 which was homozygous and fixed within the population, and which we designate 1L-57. In addition, 16/17 male specimens from Nyamongo I and 3/3 male specimens from Bayomen possessed a sex-linked band dimorphism (3C-Sp) and a heterozygous inversion (3L/82) near the centromere of chromosome 3 (Fig. 4b). This was absent in all 29 females collected from both sites. The involvement of 3C in sex determination among *S. squamosum* is diagnostic for *S. squamosum* E (= type III) [32, 33]. Since only a small number of specimens were examined from a narrow geographic range, we propose the name *S. squamosum* E2 for this variant possessing inversion 1L-57.

An additional five larval specimens collected at Bayomen were identified as *S. mengense* based on the 1CER expanded centromere of chromosome 1 (Fig. 4c) and tufts of hair-like scales on the anterior dorsum of the larval thorax (Fig. 3b) [10, 15, 44]. Adult *S. mengense* have hairs on the subcostal wing vein [10, 44], but no adult flies examined from collections made between July 2016 and January 2017 (Bayomen *n* = 254, Nyamongo I *n* = 74, and Ondouano *n* = 77) possessed this characteristic.

### Adult blackfly collections and biting rates

**Fig. 5:**
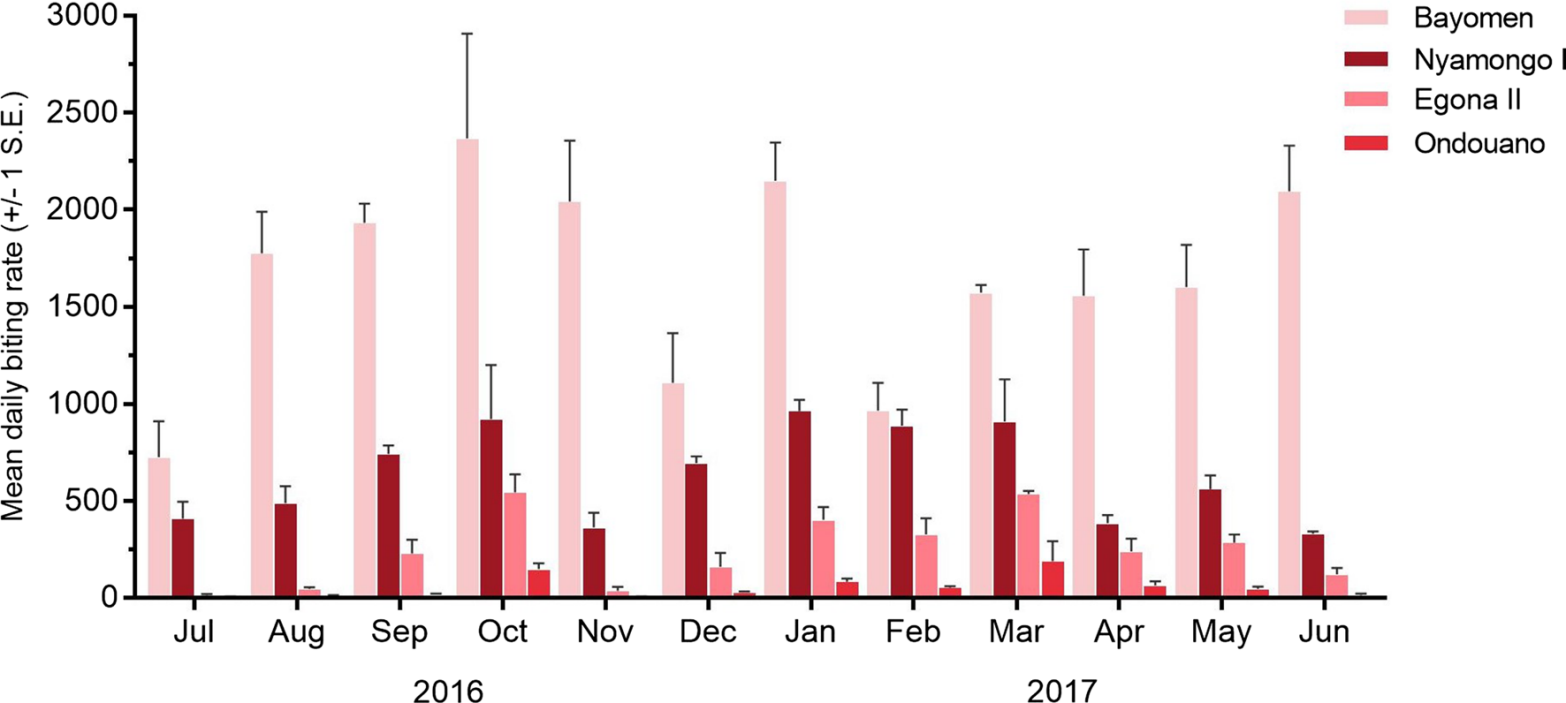
Mean daily biting rates ± 1 standard error (SE) at the four collection sites based on three collection days each month. Biting rates were highest at the two riverside sites (Bayomen and Nyamongo I) and decreased with increasing distance from the river

A total of 93,573 adult female *S. damnosum* s.l. were recorded biting humans across the four sites during the sampling period (Additional file 1: Tables S1–S4, Additional file 2: Dataset). Of the specimens collected, 9281 were dissected for parity and evidence of *Onchocerca* species infection. The remainder were preserved in ethanol for pool screening. There was some discrepancy between the number of black flies recorded on field collection forms and the actual numbers dissected and preserved for pool screening. These manual count errors were mainly caused by high blackfly densities but do not alter patterns of biting and transmission. Biting rates were highest at the two riverside sites, but a generalized negative binomial regression showed that monthly catches were significantly higher at Bayomen than Nyamongo I (*P* < 0.001) (Additional file 1: Table S5). The mean daily biting rate at Bayomen remained consistently high throughout the year and although it decreased in July, December, and February, it remained above 700 black flies/person/day (Fig. 5). Annual biting rates (ABR) of 606,370 and 233,167 were estimated at Bayomen and Nyamongo I, respectively. There were two clear peaks in biting at Nyamongo I. The first coincided with the months of highest average rainfall in September and October, before rates decreased abruptly in November at the onset of the long dry season. A second increase occurred in December, and biting rates remained high until March, before decreasing in April at the onset of the new rainy season. Most of the biting at Nyamongo I, therefore, occurred during the long dry season. Similar seasonal patterns were observed at Egona II (ABR 89,849) and Ondouano (ABR 20,540), although blackfly activity decreased with increasing distance from the river (Fig. 5). Peaks in biting were recorded at both sites in October and March, but daily biting rates at Ondouano otherwise remained relatively low (< 85). Monthly biting rates at Egona II and Ondouano were both significantly lower than at Nyamongo I (*P* < 0.001) (Additional file 1: Table S5).

### Parity rates

**Fig. 6:**
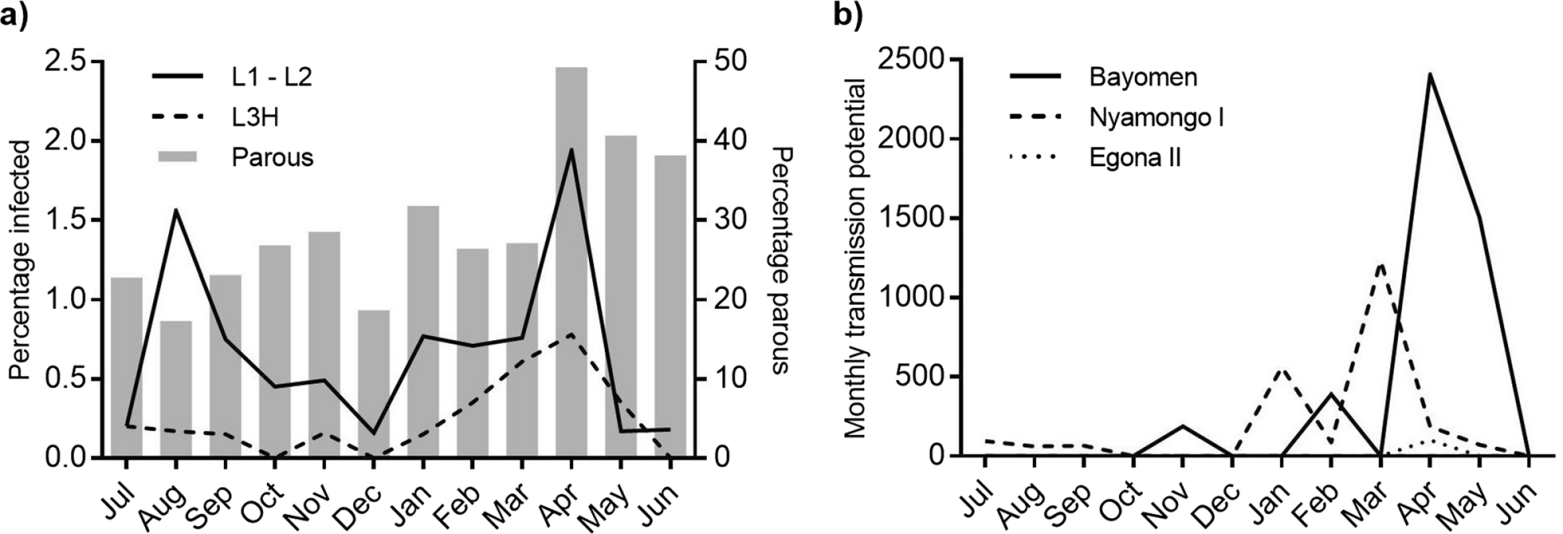
Seasonal parasite transmission along the lower Mbam river showing **a** combined parity and infection rates for flies dissected at Bayomen and Nyamongo I riverside sites (L1–L2 = percentage of flies infected with developing parasite stages only, L3H = percentage of flies containing L3 stages in the head), and **b** monthly transmission potentials at Bayomen, Nyamongo I, and Egona II estimated based on dissection data only. Ondouano not shown since no larvae were found in dissected flies

The Wilson method showed that the overall percentage of parous flies was higher at Bayomen (36.3%, 95% CI 34.7–37.8%) than at Nyamongo I (20.7%, 95% CI 19.3–22%) (*P* < 0.001) (Additional file 1: Tables S1–S2). Parity rates were lower at Egona II (11.1%, 95% CI 9.6–12.6%) (*P* < 0.001) and Ondouano (9.4%, 95% CI 0.7–12.3%) (*P* < 0.001) than at Nyamongo I, but there was no difference between Egona II and Ondouano (*P* = 0.29) (Additional file 1: Tables S3–S4). Parity rates were higher in the March–June rainy season than in the August–October rainy season based on data from both riverside sites combined (*P* < 0.001) (Fig. 6a). The odds ratio for being bitten by a parous fly was 1.8 times higher in the March–June rainy season than in the August–October rainy season. Parity rates were < 10% at Egona II and < 5% at Ondouano when biting rates peaked at these sites in October 2016 and March 2017.

### *Onchocerca* species transmission intensity as assessed by dissection

Parasites that were morphologically similar to *O. volvulus* were mainly transmitted between January and May, overlapping the dry and rainy seasons during this period. The ATP was higher and peaks in transmission occurred slightly later at Bayomen (ATP 4488) than at Nyamongo I (2360) (Fig. 6b; Additional file 1: Table S6). The mean number of L3H per infective fly was higher at Bayomen (6.4) than Nyamongo I (2.4), and the highest estimated MTP (2406) at Bayomen was calculated based on just two infective flies carrying 17 L3H parasites. When data were combined for the two riverside sites, there was no effect of rainy season on the proportion of flies carrying only L1 and L2 stage parasites (*P* = 0.528), suggesting that they were present throughout the year (Fig. 6a). However, the proportion of flies carrying L3H parasites was higher in the March–June rainy season (*P* = 0.043), corresponding with higher parity rates and suggesting that parasites were developing to transmissible forms during this period (Fig. 6a). At Egona II, the ATP (102) was substantially lower than at the riverside sites (Fig. 6b; Additional file 1: Table S6). Infective L3H parasites were only found at Egona II in two flies collected in April 2017, at the beginning of the rainy season and shortly after biting rates had peaked (Additional file 1: Table S3). No parasites of any stage were found in the 489 flies dissected at Ondouano during the study (Additional file 1: Table S4). When considering all L3 larvae, regardless of their location in the blackfly, the ATP at Bayomen increased to 5479, but all other sites remained unchanged.

### *Onchocerca* spp. transmission intensity as assessed by pool screening

**Fig. 7:**
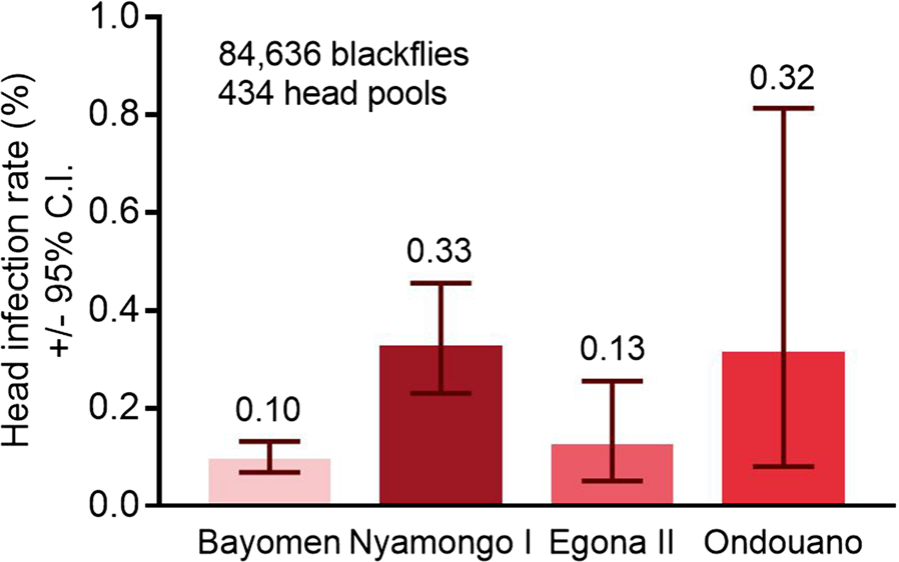
Results of pool screening showing maximum likelihood estimate of the percentage of *S. damnosum* s.l. possessing L3 larvae in their heads (± 95% CI) based on black flies collected over the 12-month sampling period. The number above the bar denotes the maximum likelihood point estimate for each site

*Onchocerca volvulus* was the predominant species developing to infective stages in *S. damnosum* s.l. (106/434 head pools), and while *O. ochengi* was present in the area (7/417 body pools), only 1/434 head pools were positive for infection. Pool screening revealed estimated infection rates in blackfly heads at Bayomen (0.10%), Nyamongo I (0.33%), and Egona II (0.13%), similar to the percentage of infective (L3H) flies encountered by dissection during the study at these sites (0.13%, 0.36%, and 0.13%, respectively) (Fig. 7). However, 4/15 pools containing 1453 black flies were positive at Ondouano, 7.9 km from the riverside, despite parasites being absent from the 489 flies dissected at this site.

## Discussion

Our study confirmed ongoing transmission of *O. volvulus* by black flies along the lower Mbam river in 2016/2017. Breeding site surveys were limited to a single period in the dry season and revealed the presence of *S. squamosum* s.l. and *S. mengense*. The involvement of chromosomal inversion 3C in sex determination and the presence of a new inversion in 1L indicates that the former is a variant of *S. squamosum* E, and not *S. squamosum* A, which was previously reported in the Mbam river basin, upstream from our study sites [4]. The E cytotype is currently known from western Côte d’Ivoire, Ghana, Guinea, Liberia, and Sierra Leone [5, 33], while previous reports of its presence in Benin, Central African Republic, and Togo were made in error [8, 33]. Our collection of *S. squamosum* E2 from the Mbam river is, therefore, the first record of this cytotype (or a variant of it) east of Lake Volta, more than 1200 km away.

While there is little reason to suggest that *S. squamosum* A was absent from the area, it is possible that the composition of cytoforms breeding along the lower Mbam has changed over time. Reductions in rainfall and river discharge have occurred since the early 1970s, and dams were built upstream from Bafia on the Noun and Mbam rivers in 1974 and 1987, respectively [24]. Environmental changes such as these alter the physical properties of river water, including rates of discharge, which could affect the blackfly species present [24, 45]. However, these events pre-date collections by Barbazan et al. [11] in 1993/1994. Another possible explanation for the different cytotypes encountered between the studies is that their composition changes seasonally. Traoré-Lamizana and Lemasson [6] have already shown that *S. squamosum* s.l. spreads north from the forest–savannah transition zones in Cameroon and into savannah rivers during the rainy season, but this was before the different *S. squamosum* cytotypes had been described. Spatiotemporal studies of the *S. squamosum* cytotypes present in Cameroon are needed to document the extent and dynamics of their distributions. Additionally, the breeding range and vector competence of *S. squamosum* E2 needs to be investigated in view of the severe clinical presentation of onchocerciasis in the Mbam valley.

We encountered high densities of biting black flies throughout the year at riverside sites in agreement with findings recently reported by Abong et al. [46] from nearby Biatsota village. Biting rates were higher at Bayomen than at Nyamongo I, possibly due to the proximity of collection sites to the nearest known breeding sites (< 300 m and ≈1 km, respectively). At Nyamongo I, seasonal biting patterns were similar to those recorded in 1993/1994 although ABRs were considerably higher in the current study (ABRs of 233,167 and 98,208, respectively) [11]. This was partly due to the extended biting peak that occurred during the long dry season compared with the same period in 1993/1994 [11]. The dry season peak at Nyamongo I occurred at a time (January–March) when the average river discharge was relatively low and stable (< 500 m^3^/s) (Fig. 2) [24]. These conditions minimise the impact of fluctuating water levels on blackfly breeding sites and may expose previously submerged oviposition substrates, leading to population increases [24, 47, 48]. Similar biting patterns were observed at Egona II and Ondouano, although biting peaked at these sites in October and March. The estimated ABR of 89,849 at Egona II was almost as high as the 98,208 estimated by Barbazan et al. [11] at the riverside in 1993/1994. However, the ABR declined markedly at Ondouano (20,540), 7.9 km from the riverside, whereas it was still 43,790 at a similar distance (7.2 km) in the previous study. In addition, Barbazan et al. had two more collection sites along the same transect at 13.5 km and 23 km, both of which had ABRs > 43,000 [11]. Considering that they reported no breeding along tributaries, it may be that black flies dispersed greater distances at the time of the earlier study. Recent population growth in villages close to the river has also increased the availability of potential blood hosts which might decrease the need for dispersal [49]. Alternatively, decreased dispersal may indicate the presence of a different *S. damnosum* cytotype with different behavioural characteristics.

Differences in the proximity of our riverside collection sites to the nearest breeding sites might also explain why parity rates were higher at Bayomen than at Nyamongo I. Duke [50] showed that the number of parous flies can decrease rapidly with increasing distance to breeding sites, and our results would appear to agree. It is, therefore, worth noting that the Bayomen collection site was approximately 1.5 km from Bayomen village, where blackfly parity rates might have been lower. Differences in the presence and relative abundance of non-human blood hosts might also affect parity rates between populations. It is well known that black flies feed on a variety of vertebrates including cattle [51, 52], and it has been shown that *O. ochengi* appears in black flies at times coinciding with cattle migrations in northern Cameroon [53]. Our blackfly pools that tested positive for *O. ochengi* were collected at Bayomen between October 2016 and April 2017, coinciding with the transhumance period in Cameroon [54]. The number of migratory cattle arriving in the area is unclear (M. Ronse pers. comm.), however, a 2015 report by the Programme National de Développement Participatif [25] revealed there to be fewer than 100 cows kept in the Kon-Yambetta canton (population 18,268) which includes Bayomen, and which may explain why only small numbers of *O. ochengi* were detected.

We documented a gradual increase in blackfly parity rates throughout the sampling period despite our dissection data being relatively limited (Fig. 6a). Combined parity rates at the two riverside sites were higher in the March–June rainy season than in the August–October rainy season, which agrees with previous results [11]. The parity rates recorded at our riverside collection sites were similar to those recently reported from Biatsota (18.9–41.4%) [46] but were lower than the 78% average reported from the Mbam/Sanaga basin in 1993/1994 [11]. Barbazan et al. also reported that there was little difference in parity between black flies on the shoreline and at sites further inland [11]. Contrastingly, we found significantly lower parity rates at Egona II and Ondouano than at the riverside. At our inland sites, parity rates decreased to < 10% during the months of peak biting indicating relatively little exposure to parous flies among these communities.

The combined results of blackfly catches, dissections, and pool screening showed that *O. volvulus* transmission occurred mostly at the riverside, although the estimated percentage of infective flies was lower than recently documented at Biatsota [46]. Based on dissections, transmission peaked slightly earlier at Nyamongo I than at Bayomen (Fig. 6b), but generally occurred between January and May, overlapping the dry and rainy seasons during this period. This is in broad agreement with Barbazan et al. [11] who documented peaks in *O. volvulus* transmission in February and March along their Ngoro transect, extending from Nyamongo I. The ATP of 3113 reported at the Ngoro riverside site was similar to our finding at Nyamongo I (ATP 2360). However, the higher biting rates that we used to calculate this ATP indicate that *O. volvulus* infection rates in black flies have decreased since the earlier study.

We found that peaks in *O. volvulus* infection coincided with peak biting rates at Nyamongo I, whereas at Bayomen, biting rates were continuously high throughout the year. Overall, 90% of all L3 larvae were found in the heads of dissected *S. damnosum* s.l., which is higher than in many other studies [55]. The high densities of biting black flies and a higher mean number of L3H per infective fly resulted in a higher estimated ATP at Bayomen than at Nyamongo I. Nevertheless, the collection site at Nyamongo I was more likely to be a point of significant human–vector contact and parasite transmission. It was adjacent to a busy but slow-moving ferry crossing where people often gathered for lengthy periods of time, while the site at Bayomen was 1.5 km from the village and accessed mainly by farmers and fishermen. Building a bridge at the ferry crossing might alleviate some of the congestion and reduce human–vector contact. Parasite transmission declined rapidly with increasing distance from the riverside, indicating that front line communities and others working on or near the river are at highest risk of exposure to infective bites. However, pool screening detected the presence of transmissible *O. volvulus* parasites at each of our collection sites, up to 7.9 km from the riverside. Importantly, the upper bound of the confidence interval calculated at each site was above 0.05% (equivalent to 1 in 2000 flies infected) and consequently above the WHO threshold for interruption of *O. volvulus* transmission [56].

At the time of our study, CDTI was taking place around July each year [2, 3]. Ivermectin is most effective at reducing the uptake of microfilariae by black flies during the first 6 months after treatment [57]. If *O. volvulus* transmission along the Mbam river was constant throughout the year, this might explain the lower transmission rates encountered between July and December based on the results of dissections. However, similar patterns of transmission were reported in the area in 1993/1994, and at additional sites along the Sanaga river [11] where *S. squamosum* B breeds and bites perennially [14]. These entomological studies were conducted at a time pre-dating ivermectin mass treatment in the Mbam valley [2, 58]. Even the earliest mass treatments (1994–1997) only covered approximately 10% of the population, and high (> 65%) therapeutic coverage was not achieved until several years after CDTI commenced [2, 3]. Accordingly, ivermectin would not have affected the results of the earlier study [11] and these patterns of transmission probably reflect true seasonal cycles. Similar seasonality has been reported for perennially breeding *S. squamosum* in Togo [59], where peaks in *O. volvulus* transmission coincided with high dry-season biting rates and high parity rates despite black flies being present throughout the year. Favourable weather conditions are also thought to increase the probability of blackfly survival and human–vector contact resulting in dry season peaks in transmission involving the forest cytoform, *S. yahense* [59, 60]. Consideration should, therefore, be given to seasonal patterns of *O. volvulus* transmission along the Mbam river when planning CDTI activities. Whereas communities normally decide on the period of ivermectin distribution, optimizing the timing of CDTI to coincide with the period prior to peak transmission may enhance the programme outcome at no additional cost [61]. Alternatively, annual moxidectin treatment might overcome the effects of seasonal transmission since it is effective at maintaining low skin microfilarial levels for more than 12 months after administration [61, 62]. Complementary treatment strategies such as seasonal larviciding during times of peak *O. volvulus* transmission [61] or slash and clear-based vector control methods [63] should also be considered in view of the high blackfly densities encountered along the Mbam.

Our study was mainly limited by problems encountered during pool screening which prevented longitudinal analyses of infection rates or estimates of transmission potentials based on our larger sample of black flies. High dilutions were needed (> 1:1000) to remove the effect of PCR inhibitors from DNA samples of specimens collected between February and June 2017, which suggests a problem with the DNA extraction. The dilution of samples to remove inhibitors also dilutes the target DNA and may have pushed the template below our detection limit. Smaller pool sizes might have reduced this source of error and will be considered in future studies. As a result, many of our inferences relied upon data obtained from a comparatively small number of dissected black flies, and our study does not take into consideration year-to-year variation in biting rates and parasite transmission. Nevertheless, our findings agree with previous investigations [11, 46] and show that *O. volvulus* transmission is sustained by high blackfly biting rates along the lower Mbam river where onchocerciasis continues to be a public health concern [3, 21, 58].

## Conclusions

After 16 years of annual CDTI in the lower Mbam valley, *O. volvulus* infection rates in black flies were above the 0.05% threshold for interruption of transmission. Although the percentage of infective black flies was relatively low, extremely high riverside biting rates were likely a major contributing factor to ongoing transmission in an area where a new variant of *S. squamosum* E is described.

## Supplementary Information

**Additional file 1:** **Table S1. **Summary of blackfly collection and dissection data from catches made at Bayomen between July 2016 and June 2017. **Table S2. **Summary of blackfly collection and dissection data from catches made at Nyamongo I between July 2016 and June 2017. **Table S3. **Summary of blackfly collection and dissection data from catches made at Egona II between July 2016 and June 2017. **Table S4. **Summary of blackfly collection and dissection data from catches made at Ondouano between July 2016 and June 2017. **Table S5. **Estimated monthly (MBR) and annual (ABR) biting rates at the four collection sites. **Table S6. **Estimated monthly (MTP) and annual (ATP) transmission potentials at the four collection sites.

**Additional file 2:** Dataset. File contains pool screen, dissection, and biting rate data.

## Data Availability

All data generated or analysed during this study are included in this published article [and its Additional files].
