# Supplementary material for: Onchocerca volvulus transmission in the Mbam valley of Cameroon following 16 years of annual community-directed treatment with ivermectin, and the description of a new cytotype of Simulium squamosum
Source: Parasit Vectors. 2021 Nov 2;14:563. doi: 10.1186/s13071-021-05072-y (PMC8561987; doi:10.1186/s13071-021-05072-y)
Supplement: Supplementary file 1 — Additional file 1: Table S1. Summary of blackfly collection and dissection data from catches made at Bayomen between July 2016 and June 2017. Table S2. Summary of blackfly collection and dissection data from catches made at Nyamongo I between July 2016 and June 2017. Table S3. Summary of blackfly collection and dissection data from catches made at Egona II between July 2016 and June 2017. Table S4. Summary of blackfly collection and dissection data from catches made at Ondouano between July 2016 and June 2017. Table S5. Estimated monthly (MBR) and annual (ABR) biting rates at the four collection sites. Table S6. Estimated monthly (MTP) and annual (ATP) transmission potentials at the four collection sites. [file 13071_2021_5072_MOESM1_ESM.docx]

Additional file 1: Supplementary information

*Onchocerca volvulus* transmission in the Mbam valley of Cameroon following 16 years of annual community-directed treatment with ivermectin, and the description of a new cytotype of *Simulium squamosum*

Adam Hendy, Meryam Krit, Kenneth Pfarr, Christine Laemmer, Jacobus De Witte, Philippe Nwane, Joseph Kamgno, Hugues C. Nana-Djeunga, Michel Boussinesq, Jean-Claude Dujardin, Rory Post, Robert Colebunders, Sarah O’Neill, Peter Enyong, Alfred K. Njamnshi

| **Bayomen** | **Jul** | **Aug** | **Sep** | **Oct** | **Nov** | **Dec** | **Jan** | **Feb** | **Mar** | **Apr** | **May** | **Jun** | **Total** |
| --- | --- | --- | --- | --- | --- | --- | --- | --- | --- | --- | --- | --- | --- |
| No. Days | 3 | 3 | 3 | 3 | 3 | 3 | 3 | 3 | 3 | 3 | 3 | 3 | **36** |
| Total Blackfly Catch | 2182 | 5326 | 5800 | 7105 | 6125 | 3327 | 6451 | 2902 | 4718 | 4671 | 4804 | 6289 | **59700** |
| No. Preserved* | 1949 | 4996 | 5470 | 6775 | 5799 | 3023 | 6121 | 2624 | 4383 | 4341 | 4474 | 5959 | **55914** |
| No. Dissected | 233 | 330 | 330 | 330 | 326 | 304 | 330 | 278 | 335 | 330 | 330 | 330 | **3786** |
| No. Parous | 52 | 56 | 104 | 129 | 117 | 68 | 133 | 114 | 88 | 186 | 192 | 134 | **1373** |
| Parous (%) | 22.3 | 17.0 | 31.5 | 39.1 | 35.9 | 22.4 | 40.3 | 41.0 | 26.3 | 56.4 | 58.2 | 40.6 | **36.3** |
| No. Flies with L1 - L3 (%) | 0 (0) | 3 (0.91) | 0 (0) | 1 (0.30) | 1 (0.31) | 0 (0) | 0 (0) | 4 (1.44) | 2 (0.60) | 8 (2.42) | 1 (0.30) | 0 (0) | **20 (0.53)** |
| No. Flies with L3H (%) | 0 (0) | 0 (0) | 0 (0) | 0 (0) | 1 (0.31) | 0 (0) | 0 (0) | 1 (0.36) | 0 (0) | 2 (0.61) | 1 (0.30) | 0 (0) | **5 (0.13)** |
| No. L1 | 0 | 10 | 0 | 0 | 2 | 0 | 0 | 6 | 2 | 17 | 0 | 0 | **37** |
| No. L2 | 0 | 10 | 0 | 3 | 0 | 0 | 0 | 8 | 1 | 7 | 0 | 0 | **29** |
| No. L3 (Total) | 0 | 0 | 0 | 0 | 1 | 0 | 0 | 4 | 0 | 24 | 10 | 0 | **39** |
| No. L3H (Head) | 0 | 0 | 0 | 0 | 1 | 0 | 0 | 4 | 0 | 17 | 10 | 0 | **32** |

**Table S1.** Summary of blackfly collection and dissection data from catches made at Bayomen between July 2016 and June 2017.

**Table S2.** Summary of blackfly collection and dissection data from catches made at Nyamongo I between July 2016 and June 2017.

| **Nyamongo I** | **Jul** | **Aug** | **Sep** | **Oct** | **Nov** | **Dec** | **Jan** | **Feb** | **Mar** | **Apr** | **May** | **Jun** | **Total** |
| --- | --- | --- | --- | --- | --- | --- | --- | --- | --- | --- | --- | --- | --- |
| No. Days | 3 | 3 | 3 | 3 | 3 | 3 | 3 | 3 | 3 | 3 | 3 | 3 | **36** |
| Total Blackfly Catch | 1228 | 1471 | 2230 | 2770 | 1089 | 2087 | 2896 | 2666 | 2729 | 1155 | 1682 | 996 | **22999** |
| No. Preserved* | 961 | 1224 | 1893 | 2440 | 797 | 1755 | 2576 | 2379 | 2408 | 970 | 1440 | 776 | **19619** |
| No. Dissected | 267 | 247 | 337 | 330 | 292 | 332 | 320 | 287 | 321 | 185 | 242 | 220 | **3380** |
| No. Parous | 62 | 44 | 50 | 48 | 59 | 51 | 74 | 35 | 90 | 68 | 41 | 76 | **698** |
| Parous (%) | 23.2 | 17.8 | 14.8 | 14.5 | 20.2 | 15.4 | 23.1 | 12.2 | 28.0 | 36.8 | 16.9 | 34.5 | **20.7** |
| No. Flies with L1 - L3 (%) | 2 (0.75) | 7 (2.83) | 6 (1.78) | 2 (0.61) | 3 (1.03) | 1 (0.30) | 6 (1.88) | 2 (0.70) | 7 (2.18) | 6 (3.24) | 2 (0.83) | 1 (0.45) | **45 (1.33)** |
| No. Flies with L3H (%) | 1 (0.37) | 1 (0.40) | 1 (0.30) | 0 (0) | 0 (0) | 0 (0) | 1 (0.31) | 1 (0.35) | 4 (1.25) | 2 (1.08) | 1 (0.41) | 0 (0) | **12 (0.36)** |
| No. L1 | 2 | 12 | 5 | 26 | 7 | 7 | 7 | 5 | 2 | 2 | 0 | 2 | **77** |
| No. L2 | 0 | 2 | 20 | 0 | 0 | 0 | 4 | 0 | 3 | 23 | 12 | 0 | **64** |
| No. L3 (Total) | 2 | 1 | 1 | 0 | 0 | 0 | 6 | 1 | 14 | 3 | 1 | 0 | **29** |
| No. L3H (Head) | 2 | 1 | 1 | 0 | 0 | 0 | 6 | 1 | 14 | 3 | 1 | 0 | **29** |

**Table S3.** Summary of blackfly collection and dissection data from catches made at Egona II between July 2016 and June 2017.

| **Egona II** | **Jul** | **Aug** | **Sep** | **Oct** | **Nov** | **Dec** | **Jan** | **Feb** | **Mar** | **Apr** | **May** | **Jun** | **Total** |
| --- | --- | --- | --- | --- | --- | --- | --- | --- | --- | --- | --- | --- | --- |
| No. Days | 3 | 3 | 3 | 3 | 3 | 3 | 3 | 3 | 3 | 3 | 3 | 3 | **36** |
| Total Blackfly Catch | 40 | 142 | 687 | 1636 | 110 | 482 | 1212 | 982 | 1611 | 720 | 859 | 370 | **8851** |
| No. Preserved* | 28 | 106 | 529 | 1387 | 90 | 394 | 984 | 883 | 1371 | 579 | 682 | 192 | **7225** |
| No. Dissected | 12 | 36 | 158 | 249 | 20 | 88 | 228 | 99 | 240 | 141 | 177 | 178 | **1626** |
| No. Parous | 4 | 6 | 20 | 23 | 1 | 5 | 31 | 20 | 11 | 27 | 11 | 21 | **180** |
| Parous (%) | 33.3 | 16.7 | 12.7 | 9.2 | 5 | 5.7 | 13.6 | 20.2 | 4.6 | 19.1 | 6.2 | 11.8 | **11.1** |
| No. Flies with L1 - L3 (%) | 0 (0) | 0 (0) | 3 (1.90) | 0 (0) | 0 (0) | 0 (0) | 0 (0) | 0 (0) | 2 (0.83) | 4 (2.84) | 0 (0) | 0 (0) | **9 (0.55)** |
| No. Flies with L3H (%) | 0 (0) | 0 (0) | 0 (0) | 0 (0) | 0 (0) | 0 (0) | 0 (0) | 0 (0) | 0 (0) | 2 (1.42) | 0 (0) | 0 (0) | **2 (0.12)** |
| No. L1 | 0 | 0 | 2 | 0 | 0 | 0 | 0 | 0 | 4 | 0 | 0 | 0 | **6** |
| No. L2 | 0 | 0 | 2 | 0 | 0 | 0 | 0 | 0 | 2 | 7 | 0 | 0 | **11** |
| No. L3 (Total) | 0 | 0 | 0 | 0 | 0 | 0 | 0 | 0 | 0 | 2 | 0 | 0 | **2** |
| No. L3H (Head) | 0 | 0 | 0 | 0 | 0 | 0 | 0 | 0 | 0 | 2 | 0 | 0 | **2** |

**Table S4.** Summary of blackfly collection and dissection data from catches made at Ondouano between July 2016 and June 2017.

| **Ondouano** | **Jul** | **Aug** | **Sep** | **Oct** | **Nov** | **Dec** | **Jan** | **Feb** | **Mar** | **Apr** | **May** | **Jun** | **Total** |
| --- | --- | --- | --- | --- | --- | --- | --- | --- | --- | --- | --- | --- | --- |
| No. Days | 3 | 3 | 3 | 3 | 3 | 3 | 3 | 3 | 3 | 3 | 3 | 3 | **36** |
| Total Blackfly Catch | 17 | 36 | 54 | 447 | 15 | 81 | 254 | 158 | 566 | 192 | 145 | 48 | **2013** |
| No. Preserved* | 13 | 18 | 43 | 332 | 10 | 53 | 168 | 91 | 483 | 165 | 118 | 30 | **1524** |
| No. Dissected | 4 | 18 | 11 | 115 | 5 | 28 | 86 | 67 | 83 | 27 | 27 | 18 | **489** |
| No. Parous | 1 | 6 | 2 | 5 | 0 | 7 | 14 | 3 | 2 | 0 | 0 | 6 | **46** |
| Parous (%) | 25.0 | 33.3 | 18.2 | 4.3 | 0.0 | 25.0 | 16.3 | 4.5 | 2.4 | 0.0 | 0.0 | 33.3 | **9.4** |
| No. Flies with L1 - L3 (%) | 0 (0) | 0 (0) | 0 (0) | 0 (0) | 0 (0) | 0 (0) | 0 (0) | 0 (0) | 0 (0) | 0 (0) | 0 (0) | 0 (0) | **0 (0)** |
| No. Flies with L3H (%) | 0 (0) | 0 (0) | 0 (0) | 0 (0) | 0 (0) | 0 (0) | 0 (0) | 0 (0) | 0 (0) | 0 (0) | 0 (0) | 0 (0) | **0 (0)** |
| No. L1 | 0 | 0 | 0 | 0 | 0 | 0 | 0 | 0 | 0 | 0 | 0 | 0 | **0** |
| No. L2 | 0 | 0 | 0 | 0 | 0 | 0 | 0 | 0 | 0 | 0 | 0 | 0 | **0** |
| No. L3 (Total) | 0 | 0 | 0 | 0 | 0 | 0 | 0 | 0 | 0 | 0 | 0 | 0 | **0** |
| No. L3H (Head) | 0 | 0 | 0 | 0 | 0 | 0 | 0 | 0 | 0 | 0 | 0 | 0 | **0** |

**Table S5.** Estimated monthly (MBR) and annual (ABR) biting rates at the four collection sites calculated following the methods of Walsh et al. [1].

| **Monthly Biting Rate (MBR)** | **2016** | | | | | | **2017** | | | | | |  |
| --- | --- | --- | --- | --- | --- | --- | --- | --- | --- | --- | --- | --- | --- |
|  | **Jul** | **Aug** | **Sep** | **Oct** | **Nov** | **Dec** | **Jan** | **Feb** | **Mar** | **Apr** | **May** | **Jun** | **ABR** |
| Bayomen | 22547 | 55035 | 58000 | 73418 | 61250 | 34379 | 66660 | 27085 | 48753 | 46710 | 49641 | 62890 | 606370 |
| Nyamongo I | 12689 | 15200 | 22300 | 28623 | 10890 | 21566 | 29925 | 24883 | 28200 | 11550 | 17381 | 9960 | 233167 |
| Egona II | 413 | 1467 | 6870 | 16905 | 1100 | 4981 | 12524 | 9165 | 16647 | 7200 | 8876 | 3700 | 89849 |
| Ondouano | 176 | 372 | 540 | 4619 | 150 | 837 | 2625 | 1475 | 5849 | 1920 | 1498 | 480 | 20540 |

**Table S6.** Estimated monthly (MTP) and annual (ATP) transmission potentials at the four collection sites calculated using biting rates and dissection data as described by Walsh et al. [1].

| **Monthly Transmission Potential (MTP)** | **2016** | | | | | | **2017** | | | | | |  |
| --- | --- | --- | --- | --- | --- | --- | --- | --- | --- | --- | --- | --- | --- |
|  | **Jul** | **Aug** | **Sep** | **Oct** | **Nov** | **Dec** | **Jan** | **Feb** | **Mar** | **Apr** | **May** | **Jun** | **ATP** |
| Bayomen | 0 | 0 | 0 | 0 | 188 | 0 | 0 | 390 | 0 | 2406 | 1504 | 0 | 4488 |
| Nyamongo I | 95 | 62 | 66 | 0 | 0 | 0 | 561 | 87 | 1230 | 187 | 72 | 0 | 2360 |
| Egona II | 0 | 0 | 0 | 0 | 0 | 0 | 0 | 0 | 0 | 102 | 0 | 0 | 102 |
| Ondouano | 0 | 0 | 0 | 0 | 0 | 0 | 0 | 0 | 0 | 0 | 0 | 0 | 0 |

1. Walsh JF, Davies JB, Le Berre R, Garms R. Standardization of criteria for assessing the effect of *Simulium* control in onchocerciasis control programmes. Trans R Soc Trop Med Hyg. 1978;72 6:675-6.
